# Impact of genomic preselection on subsequent genetic evaluations with ssGBLUP using real data from pigs

**DOI:** 10.1186/s12711-022-00727-5

**Published:** 2022-06-28

**Authors:** Ibrahim Jibrila, Jeremie Vandenplas, Jan ten Napel, Rob Bergsma, Roel F. Veerkamp, Mario P. L. Calus

**Affiliations:** 1 Animal Breeding and Genomics Group, Wageningen University & Research, PO Box 338, 6700 AH Wageningen, The Netherlands; 2 Topigs Norsvin Research Center B.V., Schoenaker 6, 6641 SZ Beuningen, The Netherlands

## Abstract

**Background:**

Empirically assessing the impact of preselection on genetic evaluation of preselected animals requires comparing scenarios that take different approaches into account, including scenarios without preselection. However, preselection is almost always performed in animal breeding programs, so it is difficult to have a dataset without preselection. Hence, most studies on preselection have used simulated datasets, and have concluded that genomic estimated breeding values (GEBV) from subsequent single-step genomic best linear unbiased prediction (ssGBLUP) evaluations are unbiased. The aim of this study was to investigate the impact of genomic preselection (GPS) on accuracy and bias in subsequent ssGBLUP evaluations, using data from a commercial pig breeding program.

**Methods:**

We used data on average daily gain during performance testing, average daily gain throughout life, backfat thickness, and loin depth from one sire line and one dam line of pigs. As these traits have different weights in the breeding goals of the two lines, we analyzed the lines separately. For each line, we implemented a reference GPS scenario that kept all available data, against which the next two scenarios were compared. We then implemented two other scenarios with additional layers of GPS by removing all animals without progeny either (i) only in the validation generation, or (ii) in all generations. We conducted subsequent ssGBLUP evaluations for each GPS scenario, using all the data remaining after implementing the GPS scenario. Accuracy and bias were computed by comparing GEBV against progeny yield deviations of validation animals.

**Results:**

Results for all traits and in both lines showed a marginal loss in accuracy due to the additional layers of GPS. Average accuracies across all GPS scenarios in the two lines were 0.39, 0.47, 0.56, and 0.60, for average daily gain during performance testing and throughout life, backfat thickness, and loin depth, respectively. Biases were largely absent, and when present, did not differ greatly between the GPS scenarios.

**Conclusions:**

We conclude that the impact of preselection on accuracy and bias in subsequent ssGBLUP evaluations of selection candidates in pigs is generally minimal. We expect this conclusion to apply for other animal breeding programs as well, since preselection of any type or intensity generally has the same effect in animal breeding programs.

**Supplementary Information:**

The online version contains supplementary material available at 10.1186/s12711-022-00727-5.

## Background

In animal breeding, parents of the next generation are often selected in multiple stages, with the initial stages of selection referred to as preselection [1–3]. Selection candidates that survive preselection are called preselected animals [1–3], and those that do not are called preculled animals [3, 4]. The aim of preselection is to reduce the costs and efforts required for animals that are not of interest to the breeding program, and it achieves this by avoiding phenotyping or further testing of preculled animals. Due to the introduction of genomic prediction [5], preselection is now mostly based on genomic estimated breeding values (GEBV) of young animals even before they have records for any trait. This type of preselection is called genomic preselection (GPS; e.g. [1, 2]). The popularity of GPS is due to genotyping becoming cheaper by the day, and to the reasonable reliabilities of GEBV (e.g. [6–8]). As genomically preculled animals have neither progeny nor records for some or all breeding goal traits, they are generally not included in subsequent genetic evaluations (i.e. genetic evaluations that come after preselection). Thus, GPS decreases the amount of information available for subsequent genetic evaluations of preselected animals. This may not only lead to loss of accuracy, but may also result in bias in the GEBV. Biased GEBV can lead to incorrect estimates of genetic trends, and incorrect ranking of animals across generations [9]. Properly assessing the impact of preselection on subsequent genetic evaluation of preselected animals requires comparison of scenarios that take different approaches into account, including a scenario without preselection. Because preselection is almost always performed in animal breeding programs, it is difficult, if not impossible, to have a scenario without preselection. This is why most studies on preselection have used simulated datasets (e.g. [1, 3, 10, 11]). These studies have shown that when a subsequent genetic evaluation of preselected animals is done using pedigree-based best linear unbiased prediction (PBLUP), preselection results in accuracy loss and bias in the estimated breeding values (EBV) of preselected animals [1, 3, 9–12]. Some of these studies [10–13] further showed that the accuracy loss and bias caused by GPS can be avoided if the information on preculled animals that was used in preselection is included in subsequent PBLUP evaluations. However, our previous studies [3, 4] have shown that when the subsequent genetic evaluation is done with single-step genomic BLUP (ssGBLUP), genomic EBV (GEBV) of preselected animals are estimated without bias. Furthermore, we showed that to avoid GPS bias in subsequent ssGBLUP evaluation of preselected animals, genotypes of their preculled sibs are only needed if not all of their parents are genotyped [4].

In our previous studies [3, 4], which were based on simulated datasets, preselection was the only possible source of bias in ssGBLUP evaluations. However, in real breeding programmes, other sources of bias in ssGBLUP evaluations may exist and are potentially difficult to control. Therefore, the impact of preselection might be confounded by the impact of these other factors. These other possible sources of bias include, among others, inaccurate or incomplete pedigree [14], inaccurately estimated additive genetic (co)variances [14], and a reference population of selectively genotyped animals [15, 16]. Although some approaches to reduce the bias caused by these factors have been developed, the bias is usually not completely eliminated in evaluations using real data (e.g. [14–16]). This may explain the observation that, in practice, GEBV obtained from ssGBLUP evaluations are sometimes biased.

Thus, the aim of this study was to investigate the impact of GPS on accuracy and bias in subsequent ssGBLUP evaluations, using data from a commercial pig breeding program in which preselection was performed. To achieve this aim, we used the full dataset as control and retrospectively implemented additional layers of GPS. The additional layers of GPS were implemented by discarding animals that did not have progeny in the data. Since GEBV were used to select parents of next generations in this breeding program, discarding animals without progeny in the dataset can be considered as additional GPS. Then, we compared results from subsequent ssGBLUP evaluations after these additional layers of GPS against results from ssGBLUP evaluation using the full available data. Our subsequent genetic evaluations only involved reevaluation of preselected animals, either with or without preculled animals in the subsequent evaluations.

## Methods

### Data

We obtained data on pig production traits (average daily gain during performance testing (ADGT), average daily gain throughout the pig’s lifetime (ADGL), backfat thickness, and loin depth) that were collected between 1970 and 2020 for one sire line and one dam line from Topigs Norsvin. These traits are part of the breeding goals of each line. However, there was more emphasis on reproduction traits than on production traits in the dam line. Details on the amount of data used in this study are in Table 1. The data were recorded on originally preselected animals (i.e. the animals preselected by Topigs Norsvin), with the sire line being much more balanced than the dam line, in terms of proportions of males and females with records per generation (the ratio of males with records to females with records is about 50:50 in the sire line and about 20:80 in the dam line). The impact of genomic preselection (GPS) was evaluated separately in the two lines, because the studied traits had different weights in the breeding goals of the two lines. Ancestors from the same line and year of birth with unknown parents were considered as a separate base population in the pedigree. Each base population was fitted as a genetic group to account for genetic trend and differences in origin and selection history [17].

**Table 1:** Data used in subsequent ssGBLUP^a^ evaluations following each preselection scenario, after quality control

Data in the subsequent ssGBLUP evaluation/preselection scenario	With records on animals in the validation generation			Without records on animals in the validation generation		
	Reference^b^	VGP^c^	MGP^d^	Reference^b^	VGP^c^	MGP^d^
Sire line (number of validation animals per trait is ± 1383)						
Number of animals in the pedigree	81,875	60,950	12,777	81,875	60,950	12,777
Number of animals with record for at least one trait^e^	75,129	54,217	6065	52,846	52,846	4694
Number of animals with genotypes	33,506	23,315	5131	33,506	23,315	5131
Number of SNP genotyped	20,550	20,963	20,926	20,550	20,963	20,926
Dam line (number of validation animals per trait is ± 2051)						
Number of animals in the pedigree	160,426	124,031	33,485	160,426	124,031	33,485
Number of animals with record for at least one trait	139,403	103,018	12,514	100,710	100,710	10,206
Number of animals with genotypes	50,895	36,369	9072	50,895	36,369	9072
Number of SNP genotyped	19,199	19,256	20,647	19,199	19,256	20,647

^a^Single-step genomic best linear unbiased prediction

^b^In the reference scenario, the subsequent ssGBLUP evaluation used the entire available data until the validation generation

^c^Validation generation preselection (VGP) scenario, in which all animals in the validation generation without progeny in the data were discarded

^d^Multi-generation preselection (MGP) scenario, in which all animals in the validation and training generations without progeny in the data were discarded

^e^About 87% and 70% of the animals in the sire and dam lines, respectively, had records for the four traits used in this study, and even larger numbers had records for any two and three traits. We decided to keep any animal with a record for at least one of the traits (92% and 87% of the animals in the sire and dam lines, respectively) because every animal in the analyses would benefit from records on relatives and records of correlated traits (see Additional file 1: Table S1), in addition to its own record on the primary trait

### Training and validation generations

For each line, we split the animals into two groups, based on a cut-off birth date. Animals born before or on the cut-off birth date were used as the training population, and animals born after the cut-off birth date were used as the validation population. This cut-off date was 31st January, 2017 for the sire line, and 31st December, 2015 for the dam line. Then, from the validation population, animals that met the following requirements were selected as validation animals: (1) none of their parents were in the validation population, and (2) the animals had phenotyped progeny. The first requirement ensured that the validation animals were from only one generation, and the second requirement enabled the comparison of the GEBV of the validation animals against their progeny yield deviations (PYD) [18]. Since records on validation animals were included in some of our subsequent evaluation scenarios (as described later), we chose to use PYD as proxy for true breeding values (TBV) because they were estimated from phenotypes that were not included in the subsequent genetic evaluations.

### Genomic data and quality control

Our genomic data included genotypes of animals for about 21,000 single nucleotide polymorphisms (SNPs) that segregated in both lines, and were distributed across the 18 porcine autosomes. The SNPs were genotyped using a custom SNP chip. We used the Plink software [19] for all quality control operations on our genomic data. For each GPS scenario (as described later) and for each line, animals and SNPs with call rates less than 90% were removed, as well as SNPs that deviated from Hardy–Weinberg equilibrium (Hardy–Weinberg equilibrium exact test p value = 10^–15^), or had a minor allele frequency lower than 0.005. Table 1 contains the summary of the pedigree, genomic and phenotypic information used in the subsequent genetic evaluations following each GPS scenario.

### Computation of precorrected phenotypes

In our genetic evaluations, we used precorrected rather than raw phenotypes as records. Animals from different lines were sometimes raised together, so they shared some fixed and non-genetic random effects. Because we studied the impact of GPS within lines, it was necessary to correct phenotypes for all non-genetic effects before the data were divided into lines. Another motivation for using precorrected phenotypes is that the additional GPS scenarios (as described in detail in the next section) could result in some classes of the non-genetic effects to be left with only one or a few animals. Thus, correcting for these effects would be less accurate compared to correcting for them before implementing our additional GPS scenarios. To compute precorrected phenotypes (${\mathrm{y}}_{\mathrm{c}})$, we first ran the following multi-trait pedigree-based animal model:

1 $${\mathbf{y}}_{\mathrm{j}}={{\mathbf{X}}_{\mathrm{j}}{\mathbf{b}}}_{\mathrm{j}}+{\mathbf{W}}_{\mathrm{j}}{\mathbf{p}}_{\mathrm{j}}+{\mathbf{Z}}_{\mathrm{j}}{\mathbf{u}}_{\mathrm{j}}+{\mathbf{e}}_{\mathrm{j}},$$

where for each trait ($\mathrm{j}$): ${\mathbf{y}}_{\mathrm{j}}$ is the vector of phenotypes; ${\mathbf{b}}_{\mathrm{j}}$ is the vector of fixed effects, with incidence matrix ${\mathbf{X}}_{\mathrm{j}}$; ${\mathbf{p}}_{\mathrm{j}}$ is the vector of non-genetic random effects, with incidence matrix ${\mathbf{W}}_{\mathrm{j}}$; ${\mathbf{u}}_{\mathrm{j}}$ is the vector of breeding values, with incidence matrix ${\mathbf{Z}}_{\mathrm{j}}$; and ${\mathbf{e}}_{\mathrm{j}}$ is the vector of residuals. The model assumed ${\mathbf{u}}_{\mathrm{j}}$ and ${\mathbf{e}}_{\mathrm{j}}$ to be normally distributed, each with a mean of zero (when ignoring the estimated genetic group effects for ${\mathbf{u}}_{\mathrm{j}}$). For all traits (and across all animals), $\mathbf{u}$ and $\mathbf{e}$ had variance–covariance matrices $\mathbf{A}\otimes \mathbf{G}$ and $\mathbf{I}\otimes \mathbf{R}$, respectively, where $\mathbf{A}$ is the pedigree relationship matrix among animals, $\mathbf{I}$ is an identity matrix with dimensions equal to the number of animals with records, and $\mathbf{G}$ and $\mathbf{R}$ are, respectively, the trait-by-trait additive genetic and residual variance–covariance matrices. Then, for each animal ($\mathrm{i}$) with a phenotype for trait $\mathrm{j}$, we computed its precorrected phenotype (${\mathrm{y}}_{\mathrm{cij}}$) as:

2 $${\mathrm{y}}_{\mathrm{cij}}={\widehat{\mathrm{u}}}_{\mathrm{ij}}+{\widehat{\mathrm{e}}}_{\mathrm{ij}}.$$

The (co)variance components used for this analysis were estimated, before separating the data into lines using the four-trait pedigree-based animal model of Eq. (1) in ASReml [20]. All computations of (G)EBV were performed using the MiXBLUP software [21]. We decided to use a pedigree-based model (instead of a single-step model) to estimate the variance components because previous studies [22, 23] showed that in populations undergoing genomic selection (as in our data), pedigree-based models estimate variance components in the pedigree founders at least as well as single-step models.

### Preselection

**Fig. 1:**
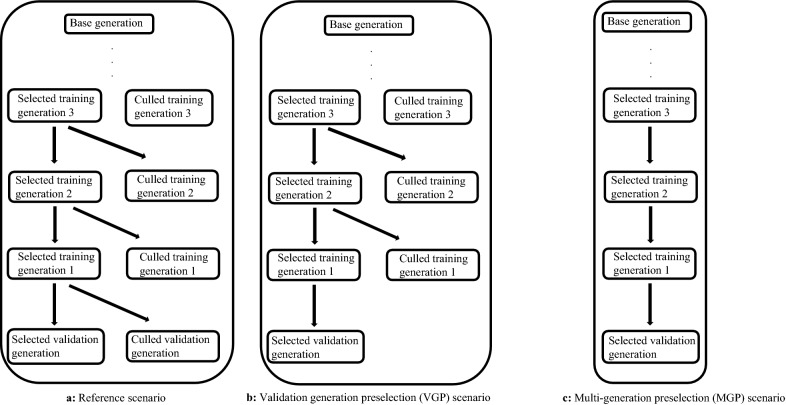
Overview of groups of animals used in subsequent ssGBLUP for each of the considered GPS scenarios

For each line, we implemented a reference scenario and two scenarios that added layers of GPS. The reference scenario—against which the other scenarios were compared—only included the original GPS implemented by Topigs Norsvin. Thus, the subsequent ssGBLUP evaluations following the reference scenario used the entire available data until the validation generation. For the second scenario, called validation generation preselection (VGP) scenario, we implemented additional GPS only in the validation generation, by discarding all animals in the validation generation that had no progeny in the data, but that did have genotypes and/or phenotypes. For the third scenario, called multi-generation preselection (MGP) scenario, we discarded any animal in the validation and training generations without progeny in the data. Animals kept after each GPS scenario are shown in Fig. 1.

### Subsequent genetic evaluations

Following each GPS scenario, we implemented a subsequent ssGBLUP evaluation with all animals that survived the GPS. We call this evaluation ‘subsequent’ because it came after the initial evaluation that provided the GEBV used in preselection. The subsequent ssGBLUP evaluations were conducted with and without records (i.e. own precorrected phenotypes) on the animals in the validation generation (see Table 1), to represent traits with records (e.g. production traits) and those without records (e.g. reproduction traits) available during subsequent evaluations. Progeny of validation animals were not included in the subsequent genetic evaluations. We estimated variance components for each preselection scenario and each line, using a pedigree-based multi-trait animal model in ASReml. We used these scenario-specific variance components in the subsequent genetic evaluations to ensure that the variance components used were appropriate for the precorrected phenotypes. In the subsequent genetic evaluations, the (multi-trait) model used for the estimations of both variance components and breeding values for each trait ($\mathrm{j}$) was:

3 $${\mathbf{y}}_{\mathbf{j}}={\mathbf 1}_{\mathbf{j}}{\upmu }_{\mathbf{j}}+{\mathbf{Z}}_{\mathbf{j}}{\mathbf{u}}_{\mathbf{j}}+{\mathbf{e}}_{\mathbf{j}},$$

where for each trait ($\mathrm{j}$): ${\mathbf{y}}_{\mathrm{j}}$ is the vector of precorrected phenotypes; ${\mathbf 1}_{\mathrm{j}}$ is an incidence vector of 1s, and ${\mathbf{Z}}_{\mathbf{j}}$ is the incidence matrix, linking precorrected phenotypes to overall mean and random animal effects, respectively; ${\upmu }_{\mathrm{j}}$ is the overall mean; ${\mathbf{u}}_{\mathrm{j}}$ is the vector of breeding values; and ${\mathbf{e}}_{\mathrm{j}}$ is the vector of residuals. The model assumed ${\mathbf{u}}_{\mathrm{j}}$ and ${\mathbf{e}}_{\mathrm{j}}$ to be normally distributed, each with a mean of zero (when ignoring the estimated genetic group effects for ${\mathbf{u}}_{\mathrm{j}}$). For all traits (and across all animals), $\mathbf{u}$ and $\mathbf{e}$ had variance–covariance matrices $\mathbf{H}\otimes \mathbf{G}$ and $\mathbf{I}\otimes \mathbf{R}$, respectively, where $\mathbf{H}$ is the combined genomic and pedigree relationship matrix among animals as explained hereafter, $\mathbf{I}$ is an identity matrix with dimensions equal to the number of animals with records, and $\mathbf{G}$ and $\mathbf{R}$ are, respectively, the trait-by-trait additive genetic and residual variance–covariance matrices. We also repeated all subsequent genetic evaluations using PBLUP, to verify the impact of using genotypes on the observed results.

### Implementation of single-step GBLUP

The inverse of the combined pedigree-genomic relationship (${\mathbf{H}}^{-1}$) was obtained as follows [24, 25]:

4 $${{\mathbf{H}}^{-1}=\mathbf{A}}^{-1}+\left[\begin{array}{cc}0& 0\\ 0& {\left(0.9{5\mathbf{G}}_{\mathbf{t}}+0.05{\mathbf{A}}_{22}\right)}^{-1}-{\mathbf{A}}_{22}^{-1}\end{array}\right],$$

where ${\mathbf{A}}^{-1}$ is the inverse of the pedigree relationship matrix, and ${\mathbf{A}}_{22}$ is part of the pedigree relationship matrix referring to genotyped animals. We considered inbreeding in the set-up of both ${\mathbf{A}}^{-1}$ and ${\mathbf{A}}_{22}$, since ignoring it has been reported to cause biases in GEBV [14]. The adjusted genomic relationship matrix ${\mathbf{G}}_{\mathbf{t}}$ was computed as follows [15, 26]:

5 $${\mathbf{G}}_{\mathbf{t}}= \left(1-{\overline{\mathrm{f}} }_{\mathrm{p}}\right){\mathbf{G}}_{\mathbf{r}}+2{\overline{\mathrm{f}} }_{\mathrm{p}}{\mathbf {11}}^{\mathbf{^{\prime}}},$$

where ${\overline{\mathrm{f}} }_{\mathrm{p}}$ is the average pedigree inbreeding coefficient across genotyped animals, ${\mathbf{G}}_{\mathbf{r}}$ is the raw genomic relationship matrix computed following the first method of VanRaden [27], and ${\mathbf {11}}^{\boldsymbol{^{\prime}}}$ is a matrix of 1s. Since in this dataset the animals with genotypes were selectively genotyped, this transformation made sure that the impact of selective genotyping was taken care of and that $\mathbf{G}$ and ${\mathbf{A}}_{22}$ were on the same scale and therefore compatible [15, 16]. To compute ${\mathbf{G}}_{\mathbf{r}}$, (current) allele frequencies were estimated using all available genomic data after quality control. We gave a weight of 0.95 to ${\mathbf{G}}_{\mathbf{t}}$ and of 0.05 to ${\mathbf{A}}_{22}$ to ensure that $\mathbf{G}$ was invertible [24, 25].

### Measures of accuracy and bias in the subsequent genetic evaluations

We used progeny yield deviation (PYD) [18] as a proxy for true breeding value (TBV), against which GEBV were compared when computing accuracy and bias. To compute PYD, we ran a multi-trait pedigree-based animal model for each line in MiXBLUP, with precorrected phenotypes as records and an overall mean as the only fixed effect (Eq. 3). The (co)variance components used in this model were also estimated for each line in ASReml, from precorrected phenotypes, using a multi-trait pedigree-based animal model that only included a mean fixed effect (Eq. 3). From the output of this analysis, we computed PYD for all validation sires and dams ($\mathrm{i}$) for each trait ($\mathrm{j}$) as:

6 $${\mathrm{PYD}}_{\mathrm{ij}}=\frac{\sum_{\mathrm{p}=1}^{\mathrm{n}}{{\mathrm{y}}_{\mathrm{cpj}}-\frac{1}{2}\mathrm{a}}_{\mathrm{mj}}}{\mathrm{n}},$$

where ${\mathrm{PYD}}_{\mathrm{ij}}$ is the PYD of a sire or dam $\mathrm{i}$ for trait $\mathrm{j}$, ${\mathrm{y}}_{\mathrm{cpj}}$ is the precorrected phenotype of a progeny $\mathrm{p}$ of the sire or dam $\mathrm{i}$ for trait $\mathrm{j}$, ${\mathrm{a}}_{\mathrm{mj}}$ is the breeding value of the mate of sire or dam $\mathrm{i}$ (for trait $\mathrm{j}$) in producing offspring $\mathrm{p}$, and $\mathrm{n}$ is the number of phenotyped progeny of sire or dam $\mathrm{i}$. Estimation of PYD was done before removing progeny of validation animals from the data. Since progeny of validation animals were not included in subsequent genetic evaluations, comparing (G)EBV to PYD can be considered as a forward-in-time validation. We computed the approximate reliability of PYD for each validation animal for each trait, and used this approximate reliability as the weighting factor for computing accuracy and bias, to account for different numbers of progeny used to estimate PYD for different validation animals. The reliability of PYD was approximated as:

7 $$\frac{1/4{\mathrm{nh}}^{2}}{1+1/4\left(\mathrm{n}-1\right){\mathrm{h}}^{2}},$$

where $\mathrm{n}$ is the validation animal’s number of half-sib progeny with records, and ${\mathrm{h}}^{2}$ is the heritability of the trait [28]. For convenience, we assumed that all the progeny of a validation animal were half-sibs, although some were full-sibs. We also computed unweighted accuracy and bias, and accuracy and bias weighted by approximate PYD reliabilities obtained when considering all progeny as full sibs. We did not observe statistically significant differences between the results, so we decided to report only the accuracy and bias weighted by approximate reliability computed when considering all progeny as half sibs (as in Eq. (7)).

Validation accuracy was computed as a weighted Pearson’s correlation coefficient between PYD and GEBV of all validation animals, using the ‘cor.test’ function of the ‘stats’ package in R [29]. We computed the standard errors (SE) of the estimates from the confidence intervals (CI) produced by the ‘cor.test’ function. Validation accuracy is not numerically the same as the accuracy of predicting TBV, since PYD have a non-genetic component, in addition to TBV [18]. However, validation accuracy and accuracy of predicting TBV increase and decrease together [30], and this property of validation accuracy enabled us to compare the subsequent genetic evaluation scenarios.

We computed two types of bias. The first type is level bias, which is a measure of whether estimated genetic gain is equal to true genetic gain. Level bias was computed as the weighted mean difference between PYD and half of the (G)EBV across all validation animals, expressed in additive genetic standard deviation (SD) units of the trait. We used the ‘weighted.mean’ function of the ‘stats’ package in R [29] to compute estimates of the weighted mean differences, and the ‘weighted_se’ function of the ‘diagis’ package in R [31] to compute SE of the estimates. A negative difference means that the GEBV were on average overestimated, and therefore genetic gain was overestimated, and vice versa. Since PYD were computed from a dataset that included information on progeny of validation animals and (G)EBV were computed without information on progeny of validation animals, PYD and (G)EBV were on different scales. Therefore, before computing differences between PYD and half of the (G)EBV of validation animals, we scaled PYD and (G)EBV to be expressed against the same genetic base, consisting of the first three training generations, by the following steps. From the model used to compute PYD, we computed average EBV across all animals in the first three training generations; next, we subtracted half of this average EBV from PYD of each validation animal; then, for each subsequent genetic evaluation, we computed the average (G)EBV of all animals in the first three training generations; and finally, we subtracted this average (G)EBV from (G)EBV of each validation animal.

The second type of bias that we computed is dispersion bias, which is a measure of how correctly relative differences in (G)EBV between animals are estimated, i.e. whether the scale of (G)EBV is correct. Dispersion bias was measured by the weighted regression coefficient of PYD on (G)EBV for all validation animals. We used the ‘lm’ function of the ‘stats’ package in R [29] to compute both the estimates and SE of the regression coefficients. If the regression coefficient is equal to its expected value, then there is no dispersion bias, and differences in (G)EBV of animals are correctly scaled. A regression coefficient lower than the expected value means that differences in (G)EBV between animals are inflated, and a regression coefficient higher than the expected value means that differences in (G)EBV between animals are deflated. In this study, the expected value of the regression coefficient is 0.5, because PYD only includes half of the breeding value of a parent.

## Results

Table 2 shows the means and SD of precorrected phenotypes of the traits analyzed, after implementation of the additional layers of genomic preselection (GPS). The results show that implementation of GPS was effective, as the lines were selected (and preselected) for increased feed efficiency (i.e. slightly increased feed intake and highly increased average daily gain), slightly decreased backfat thickness, and slightly increased loin depth. Since the validation generation for both the validation generation preselection (VGP) and multi-generation preselection (MGP) scenarios contained only the preselected animals, the means and SD of precorrected phenotypes of the traits are the same for these two GPS scenarios when only considering the validation animals. When only the validation generation was considered (i.e. the middle part of Table 2), the means of precorrected phenotypes of ADGT and ADGL increased from the reference scenario to the VGP and MGP scenarios. At the same time, the SD of precorrected phenotypes of these traits decreased from the reference scenario to the VGP and MGP scenarios. In other words, the means of precorrected phenotypes of these traits were larger and the SD of the precorrected phenotypes were lower for the preselected animals in the validation generation than for all the animals in the validation generation. For backfat thickness, both the mean and SD decreased slightly from the reference to the VGP and MGP scenarios. For loin depth, the change was also limited, with a slightly increasing mean and a slightly decreasing SD, from the reference to the VGP and MGP scenarios. MGP was more effective than VGP as shown in the right part of Table 2 (i.e. when the means and SD were computed across the entire data). For the positively (pre)selected traits (i.e. ADGT, ADGL, and loin depth), the means were higher for MGP than for VGP. For backfact, which was negatively (pre)selected, the mean was lower for MGP than for VGP. As expected, SD were in all cases lower for MGP than for VGP.

**Table 2:** Means and SD (in brackets)^a^ of precorrected phenotypes of the traits used in this study after each GPS scenario

Trait/preselection scenario	Within the validation generation only		Across the entire dataset	
	Reference^b^	VGP^c^/MGP^d^	VGP	MGP
Sire line				
ADGT (g/day)	0.26 (2.01)	1.03 (1.59)	− 0.17 (2.05)	0.77 (1.71)
ADGL (g/day)	0.07 (1.97)	0.81 (1.78)	− 0.21 (1.95)	0.61 (1.67)
Backfat thickness (mm)	− 0.32 (1.20)	− 0.35 (1.15)	− 0.02 (1.32)	− 0.13 (1.25)
Loin depth (mm)	0.34 (1.32)	0.35 (1.29)	0.23 (1.34)	0.27 (1.30)
Dam line				
ADGT (g/day)	0.40 (1.87)	1.16 (1.69)	− 0.12 (1.75)	0.71 (1.60)
ADGL (g/day)	0.21 (1.84)	0.98 (1.61)	− 0.19 (1.81)	0.58 (1.62)
Backfat thickness (mm)	0.10 (1.32)	0.06 (1.28)	− 0.03 (1.43)	− 0.09 (1.41)
Loin depth (mm)	0.31 (1.42)	0.19 (1.39)	0.28 (1.39)	0.33 (1.37)

*ADGT* average daily gain during performance testing, *ADGL* ADG throughout life

^a^Both means and SD are in additive genetic SD units

^b^In the reference scenario, the subsequent ssGBLUP evaluation utilized the entire available data until the validation generation

^c^Validation generation preselection (VGP) scenario, in which all animals in the validation generation without progeny in the data were discarded

^d^Multi-generation preselection (MGP) scenario, in which all animals in the validation and training generations without progeny in the data were discarded

The results of the subsequent genetic evaluations conducted with ssGBLUP and with PBLUP are in Tables 3 and 4 and Tables 5 and 6, respectively. For each parameter in these tables (i.e. estimated heritability, validation accuracy, level bias, and dispersion bias), both the estimate and its SE are provided. A one-tailed two-sample t-test at a 5% significance level was used to determine whether two estimates were different. We included the estimated heritabilities in our results because they help explain the results for accuracy and bias.

**Table 3:** Performance of ssGBLUP^a^ in the subsequent evaluations in the sire line (SE in brackets)

Measure/preselection scenario	With records on animals in the validation generation			Without records on animals in the validation generation		
	Reference^b^	VGP^c^	MGP^d^	Reference	VGP	MGP
Average daily gain during performance testing, size of validation population = 1382						
Estimated heritability^e^	0.24 (0.01)	0.25 (0.01)	0.33 (0.02)	0.24 (0.01)	0.24 (0.01)	0.35 (0.03)
Validation accuracy^f^	0.51 (0.02)	0.51 (0.02)	0.50 (0.02)	0.47 (0.02)	0.47 (0.02)	0.44 (0.02)
Level bias^g^	− 0.09 (0.02)	− 0.15 (0.02)	− 0.01 (0.02)	− 0.11(0.02)	− 0.11(0.02)	− 0.02 (0.02)
Dispersion bias^h^	0.48 (0.02)	0.49 (0.02)	0.48 (0.02)	0.48 (0.02)	0.48 (0.02)	0.46 (0.03)
Average daily gain throughout life, size of validation population = 1383						
Estimated heritability	0.26 (0.01)	0.28 (0.01)	0.33 (0.03)	0.27 (0.01)	0.27 (0.01)	0.35 (0.03)
Validation accuracy	0.57 (0.02)	0.56 (0.02)	0.55 (0.02)	0.52 (0.02)	0.52 (0.02)	0.48 (0.02)
Level bias	− 0.10 (0.02)	− 0.17 (0.02)	− 0.06 (0.02)	− 0.14 (0.02)	− 0.14 (0.02)	− 0.08 (0.02)
Dispersion bias	0.48 (0.02)	0.49 (0.02)	0.50 (0.02)	0.47 (0.02)	0.47 (0.02)	0.49 (0.02)
Backfat thickness, size of validation population = 1383						
Estimated heritability	0.58 (0.01)	0.58 (0.01)	0.58 (0.02)	0.58 (0.01)	0.58 (0.01)	0.60 (0.03)
Validation accuracy	0.69 (0.01)	0.68 (0.01)	0.67 (0.01)	0.63 (0.02)	0.63 (0.02)	0.56 (0.02)
Level bias	− 0.02 (0.01)	− 0.03 (0.01)	− 0.03 (0.01)	− 0.05 (0.01)	− 0.05 (0.01)	− 0.09 (0.01)
Dispersion bias	0.48 (0.01)	0.47 (0.01)	0.47 (0.01)	0.44 (0.01)	0.44 (0.01)	0.42 (0.02)
Loin depth, size of validation population = 1383						
Estimated heritability	0.55 (0.01)	0.55 (0.01)	0.55 (0.03)	0.55 (0.01)	0.55 (0.01)	0.57 (0.03)
Validation accuracy	0.68 (0.01)	0.67 (0.01)	0.65 (0.02)	0.62 (0.02)	0.62 (0.02)	0.54 (0.02)
Level bias	0.01 (0.01)	0.00 (0.01)	0.00 (0.01)	0.00 (0.01)	0.00 (0.01)	− 0.01 (0.01)
Dispersion bias	0.50 (0.01)	0.50 (0.01)	0.48 (0.02)	0.48 (0.02)	0.48 (0.02)	0.45 (0.02)

^a^Single-step genomic best linear unbiased prediction

^b^In the reference scenario, the subsequent ssGBLUP evaluation used the entire available data until the validation generation

^c^Validation generation preselection (VGP) scenario, in which all animals in the validation generation without progeny in the data were discarded

^d^Multi-generation preselection (MGP) scenario, in which all animals in the validation and training generations without progeny in the data were discarded

^e^The heritability was estimated from an equivalent pedigree-based animal model in ASReml

^f^Validation accuracy was computed as weighted Pearson’s correlation coefficient between progeny yield deviation and genomic estimated breeding value of all validation animals

^g^Level bias was computed as the weighted mean difference between progeny yield deviation and half of the genomic estimated breeding value across all validation animals, expressed in additive genetic standard deviation units of the trait

^h^Dispersion bias was measured by the weighted regression coefficient of progeny yield deviation on genomic estimated breeding value of all validation animals

**Table 4:** Performance of ssGBLUP^a^ in the subsequent evaluations in the dam line (SE in brackets)

Measure/preselection scenario	With records on animals in the validation generation			Without records on animals in the validation generation		
	Reference^b^	VGP^c^	MGP^d^	Reference	VGP	MGP
Average daily gain during performance testing, size of validation population = 2323						
Estimated heritability^e^	0.31 (0.01)	0.32 (0.01)	0.40 (0.02)	0.30 (0.01)	0.30 (0.01)	0.38 (0.02)
Validation accuracy^f^	0.35 (0.02)	0.31 (0.02)	0.29 (0.02)	0.28 (0.02)	0.28 (0.02)	0.23 (0.02)
Level bias^g^	− 0.05 (0.02)	− 0.14 (0.02)	0.04 (0.02)	0.03 (0.02)	0.03 (0.02)	0.14 (0.02)
Dispersion bias^h^	0.46 (0.03)	0.43 (0.03)	0.41 (0.03)	0.44 (0.03)	0.44 (0.03)	0.43 (0.04)
Average daily gain throughout life, size of validation population = 2405						
Estimated heritability	0.31 (0.01)	0.33 (0.01)	0.43 (0.02)	0.31 (0.01)	0.31 (0.01)	0.44 (0.02)
Validation accuracy	0.46 (0.02)	0.42 (0.02)	0.42 (0.02)	0.38 (0.02)	0.38 (0.02)	0.35 (0.02)
Level bias	− 0.06 (0.01)	− 0.16 (0.01)	− 0.01 (0.01)	0.00 (0.01)	0.00 (0.01)	0.08 (0.01)
Dispersion bias	0.45 (0.02)	0.42 (0.02)	0.42 (0.02)	0.43 (0.02)	0.43 (0.02)	0.43 (0.02)
Backfat thickness thickness, size of validation population = 2312						
Estimated heritability	0.51 (0.01)	0.51 (0.01)	0.51 (0.02)	0.51 (0.01)	0.51 (0.01)	0.53 (0.02)
Validation accuracy	0.52 (0.01)	0.50 (0.02)	0.50 (0.02)	0.45 (0.02)	0.45 (0.02)	0.42 (0.02)
Level bias	0.02 (0.01)	− 0.01 (0.01)	− 0.03 (0.01)	0.02 (0.01)	0.02 (0.01)	− 0.01 (0.01)
Dispersion bias	0.43 (0.01)	0.41 (0.01)	0.41 (0.01)	0.42 (0.02)	0.42 (0.02)	0.41 (0.02)
Loin depth, size of validation population = 1164						
Estimated heritability	0.50 (0.01)	0.50 (0.01)	0.55 (0.02)	0.49 (0.01)	0.49 (0.01)	0.53 (0.02)
Validation accuracy	0.62 (0.02)	0.60 (0.02)	0.59 (0.02)	0.55 (0.02)	0.56 (0.02)	0.49 (0.02)
Level bias	− 0.02 (0.02)	− 0.03 (0.02)	0.02 (0.02)	− 0.04 (0.02)	− 0.04 (0.02)	0.03 (0.02)
Dispersion bias	0.54 (0.02)	0.54 (0.02)	0.52 (0.02)	0.53 (0.02)	0.53 (0.02)	0.51 (0.03)

^a^Single-step genomic best linear unbiased prediction

^b^In the reference scenario, the subsequent ssGBLUP evaluation utilized the entire available data until the validation generation

^c^Validation generation preselection (VGP) scenario, in which all animals in the validation generation without progeny in the data were discarded

^d^Multi-generation preselection (MGP) scenario, in which all animals in the validation and training generations without progeny in the data were discarded

^e^The heritability was estimated from an equivalent pedigree-based animal model in ASReml

^f^Validation accuracy was computed as weighted Pearson’s correlation coefficient between progeny yield deviation and genomic estimated breeding value of all validation animals

^g^Level bias was computed as the weighted mean difference between progeny yield deviation and half of the genomic estimated breeding value across all validation animals, expressed in additive genetic standard deviation units of the trait

^h^Dispersion bias was measured by the weighted regression coefficient of progeny yield deviation on genomic estimated breeding value of all validation animals

**Table 5:** Performance of PBLUP^a^ in the subsequent evaluations in the sire line (SE in brackets)

Measure/preselection scenario	With records on animals in the validation generation			Without records on animals in the validation generation		
	Reference^b^	VGP^c^	MGP^d^	Reference	VGP	MGP
Average daily gain during performance testing, size of validation population = 1382						
Estimated heritability	0.24 (0.01)	0.25 (0.01)	0.33 (0.02)	0.24 (0.01)	0.24 (0.01)	0.35 (0.03)
Validation accuracy^e^	0.51 (0.02)	0.50 (0.02)	0.49 (0.02)	0.41 (0.02)	0.41 (0.02)	0.40 (0.02)
Level bias^f^	− 0.04 (0.02)	− 0.11 (0.02)	0.01 (0.02)	− 0.01 (0.02)	− 0.01 (0.02)	0.01 (0.02)
Dispersion bias^g^	0.53 (0.02)	0.54 (0.03)	0.48 (0.02)	0.55 (0.03)	0.55 (0.03)	0.49 (0.03)
Average daily gain throughout life, size of validation population = 1383						
Estimated heritability	0.26 (0.01)	0.28 (0.01)	0.33 (0.03)	0.27 (0.01)	0.27 (0.01)	0.35 (0.03)
Validation accuracy	0.58 (0.02)	0.56 (0.02)	0.54 (0.02)	0.47 (0.02)	0.47 (0.02)	0.44 (0.02)
Level bias	− 0.06 (0.02)	− 0.14 (0.02)	− 0.04 (0.02)	− 0.05 (0.02)	− 0.05 (0.02)	− 0.05 (0.02)
Dispersion bias	0.55 (0.02)	0.55 (0.02)	0.51 (0.02)	0.56 (0.03)	0.56 (0.03)	0.54 (0.03)
Backfat thickness thickness, size of validation population = 1383						
Estimated heritability	0.58 (0.01)	0.58 (0.01)	0.58 (0.02)	0.58 (0.01)	0.58 (0.01)	0.60 (0.03)
Validation accuracy	0.67 (0.01)	0.66 (0.02)	0.66 (0.02)	0.48 (0.02)	0.48 (0.02)	0.46 (0.02)
Level bias	− 0.03 (0.01)	− 0.03 (0.01)	− 0.03 (0.01)	− 0.09 (0.01)	− 0.09 (0.01)	− 0.10 (0.01)
Dispersion bias	0.50 (0.01)	0.50 (0.02)	0.50 (0.02)	0.46 (0.02)	0.46 (0.02)	0.43 (0.02)
Loin depth, size of validation population = 1383						
Estimated heritability	0.55 (0.01)	0.55 (0.01)	0.55 (0.03)	0.55 (0.01)	0.55 (0.01)	0.57 (0.03)
Validation accuracy	0.66 (0.02)	0.65 (0.02)	0.64 (0.02)	0.49 (0.02)	0.49 (0.02)	0.46 (0.02)
Level bias	0.00 (0.01)	0.00 (0.01)	0.00 (0.01)	0.01 (0.01)	0.01 (0.01)	0.00 (0.01)
Dispersion bias	0.50 (0.02)	0.49 (0.02)	0.49 (0.02)	0.48 (0.02)	0.48 (0.02)	0.46 (0.02)

^a^Pedigree-based best linear unbiased prediction

^b^In the reference scenario, the subsequent PBLUP evaluation utilized the entire available data until the validation generation

^c^Validation generation preselection (VGP) scenario, in which all animals in the validation generation without progeny in the data were discarded

^d^Multi-generation preselection (MGP) scenario, in which all animals in the validation and training generations without progeny in the data were discarded

^e^Validation accuracy was computed as weighted Pearson’s correlation coefficient between progeny yield deviation and estimated breeding value of all validation animals

^f^Level bias was computed as the weighted mean difference between progeny yield deviation and half of the estimated breeding value across all validation animals, expressed in additive genetic standard deviation units of the trait

^g^Dispersion bias was measured by the weighted regression coefficient of progeny yield deviation on estimated breeding value of all validation animals

**Table 6:** Performance of PBLUP^a^ in the subsequent evaluations in the dam line (SE in brackets)

Measure/preselection scenario	With records on animals in the validation generation			Without records on animals in the validation generation		
	Reference^b^	VGP^c^	MGP^d^	Reference	VGP	MGP
Average daily gain during performance testing, size of validation population = 2323						
Estimated heritability	0.31 (0.01)	0.32 (0.01)	0.40 (0.02)	0.30 (0.01)	0.30 (0.01)	0.38 (0.02)
Validation accuracy^e^	0.35 (0.02)	0.30 (0.02)	0.30 (0.02)	0.24 (0.02)	0.24 (0.02)	0.21 (0.02)
Level bias^f^	− 0.04 (0.02)	− 0.16 (0.02)	0.01 (0.02)	0.08 (0.02)	0.08 (0.02)	0.13 (0.02)
Dispersion bias^g^	0.52 (0.03)	0.45 (0.03)	0.42 (0.03)	0.50 (0.04)	0.50 (0.04)	0.45 (0.04)
Average daily gain throughout life, size of validation population = 2405						
Estimated heritability	0.31 (0.01)	0.33 (0.01)	0.43 (0.02)	0.31 (0.01)	0.31 (0.01)	0.44 (0.02)
Validation accuracy	0.48 (0.01)	0.43 (0.02)	0.43 (0.02)	0.34 (0.02)	0.34 (0.02)	0.31 (0.02)
Level bias	− 0.05 (0.01)	− 0.18 (0.01)	− 0.03 (0.01)	0.05 (0.02)	0.05 (0.02)	0.07 (0.01)
Dispersion bias	0.51 (0.02)	0.47 (0.02)	0.44 (0.02)	0.51 (0.03)	0.51 (0.03)	0.44 (0.03)
Backfat thickness thickness, size of validation population = 2312						
Estimated heritability	0.51 (0.01)	0.51 (0.01)	0.51 (0.02)	0.51 (0.01)	0.51 (0.01)	0.53 (0.02)
Validation accuracy	0.52 (0.02)	0.50 (0.02)	0.50 (0.02)	0.37 (0.02)	0.37 (0.02)	0.36 (0.02)
Level bias	0.02 (0.01)	0.00 (0.01)	− 0.03 (0.01)	0.04 (0.01)	0.04 (0.01)	0.00 (0.01)
Dispersion bias	0.45 (0.02)	0.43 (0.02)	0.42 (0.02)	0.41 (0.02)	0.41 (0.02)	0.39 (0.02)
Loin depth, size of validation population = 1164						
Estimated heritability	0.50 (0.01)	0.50 (0.01)	0.55 (0.02)	0.49 (0.01)	0.49 (0.01)	0.53 (0.02)
Validation accuracy	0.58 (0.02)	0.56 (0.02)	0.56 (0.02)	0.43 (0.02)	0.43 (0.02)	0.41 (0.02)
Level bias	0.00 (0.02)	− 0.01 (0.02)	0.04 (0.02)	− 0.02 (0.02)	− 0.02 (0.02)	0.04 (0.02)
Dispersion bias	0.55 (0.02)	0.54 (0.02)	0.51 (0.02)	0.57 (0.03)	0.57 (0.03)	0.52 (0.03)

^a^Pedigree-based best linear unbiased prediction

^b^In the reference scenario, the subsequent PBLUP evaluation utilized the entire available data until the validation generation

^c^Validation generation preselection (VGP) scenario, in which all animals in the validation generation without progeny in the data were discarded

^d^Multi-generation preselection (MGP) scenario, in which all animals in the validation and training generations without progeny in the data were discarded

^e^Validation accuracy was computed as weighted Pearson’s correlation coefficient between progeny yield deviation and estimated breeding value of all validation animals

^f^Level bias was computed as the weighted mean difference between progeny yield deviation and half of the estimated breeding value across all validation animals, expressed in additive genetic standard deviation units of the trait

^g^Dispersion bias was measured by the weighted regression coefficient of progeny yield deviation on estimated breeding value of all validation animals

For both lines, the estimated heritabilities for ADGT and ADGL did not differ between the reference and VGP scenarios, but increased in the MGP scenarios. For backfat thickness, heritability estimates did not differ between the GPS scenarios, neither in the sire line nor in the dam line. For loin depth, heritability estimates did not differ between GPS scenarios in the sire line, but in the dam line they were higher in the MGP scenarios than in the reference and VGP scenarios. For all traits, the above trends in heritability estimates were observed regardless of whether records on animals in the validation generation were included or not when estimating heritabilities. Without records on animals in the validation generation in the subsequent ssGBLUP evaluations, all results (including estimated heritabilities) for the reference and VGP scenarios were the same. The increases in heritabilities observed with additional layers of preselection were generally due to decreases in residual variances with additional layers of preselection, while additive genetic variances generally did not differ between GPS scenarios (see Additional file 2: Table S2 and Additional file 3: Table S3).

### Subsequent ssGBLUP evaluations with records on validation animals

Validation accuracies did not differ between GPS scenarios for all traits in both lines, except for ADGT in the dam line, for which the accuracy was lower in the MGP scenario than in the reference scenario (Table 4). Tendencies (i.e. indications that may not be statistically significant) towards lower accuracies with more GPS were however observed for all traits in both lines (Tables 3, 4). In both lines, level bias was absent in all scenarios for loin depth, and only in some scenarios for ADGT, ADGL and backfat thickness. Even when level bias was present, it remained marginal. The highest value of level bias recorded was -0.17 additive genetic SD units, under the VGP scenario for ADGL in the sire line (Table 3). Dispersion bias was absent (i.e. the regression coefficient of PYD on GEBV did not differ from its expected value of 0.5) for all traits in the sire line (Table 3), except in the VGP and MGP scenarios for backfat thickness, where there was inflation (i.e. the regression coefficient was less than 0.5). However, dispersion bias was present for all traits in the dam line (Table 4), except in the reference scenario for ADGT and the MGP scenario for loin depth. When dispersion bias was present in the dam line, the regression coefficients were higher than 0.5 (i.e. they were deflated) in the reference and VGP scenarios for loin depth, and lower than 0.5 in all other cases. However, the estimates of the regression coefficients did not differ across GPS scenarios within traits and lines (Tables 3, 4), although they tended to decrease from the reference to the VGP to the MGP scenarios.

### Subsequent ssGBLUP evaluations without records on validation animals

For ADGT, validation accuracy did not differ between GPS scenarios in the sire line (Table 3), but in the dam line it was lower in the MGP scenario than in the reference/VGP scenarios (Table 4). For ADGL, validation accuracies did not differ between GPS scenarios in both lines, but only tended to decrease from the reference/VGP to the MGP scenarios (Tables 3, 4). For backfat thickness, validation accuracy in the sire line decreased from the reference/VGP scenarios to the MGP scenario, but did not differ between GPS scenarios in the dam line. For loin depth, validation accuracies in both lines decreased from the reference/VGP to the MGP scenarios. Level bias was present in the reference and VGP scenarios for ADGT in the sire line, but absent in the MGP scenario, while in the dam line, level bias was absent in the reference and VGP scenarios for ADGT, and present in the MGP scenario. For ADGL, level bias was present in all GPS scenarios in the sire line, but only present in the MGP scenario in the dam line. For backfat thickness, level bias was present for all GPS scenarios in the sire line, but absent for all GPS scenarios in the dam line. For loin depth, level bias was absent for all GPS scenarios in both lines. Although level bias was present in many scenarios, it remained marginal, with ± 0.14 additive genetic SD units being the highest estimate (Tables 3, 4). For ADGT and ADGL, for all GPS scenarios dispersion bias was absent in the sire line but present in the dam line. For backfat thickness, dispersion bias was present for all scenarios in both lines. For loin depth, dispersion bias was absent for all scenarios in both lines, except for the MGP scenario in the sire line, where there was inflation. Here also, as when records on validation animals were included in the subsequent evaluations, the estimates of the regression coefficients did not differ between GPS scenarios within traits, and this was observed for all traits in both lines.

### Subsequent genetic evaluations with PBLUP

When records on animals in the validation generation were included in the subsequent evaluations, the corresponding validation accuracies did not differ between PBLUP and ssGBLUP for all traits and in both lines. However, when records on animals in the validation generation were excluded from the subsequent evaluations in the sire line, validation accuracies of all traits were lower with PBLUP than with ssGBLUP. When records on animals in the validation generation were excluded from the subsequent evaluations in the dam line, validation accuracies did not differ between PBLUP and ssGBLUP for ADGT and ADGL, but were lower with PBLUP than with ssGBLUP for backfat thickness and loin depth. As for the validation accuracies from subsequent ssGBLUP evaluations, in most cases, the validation accuracies from subsequent PBLUP evaluations did not differ between corresponding GPS scenarios, and level bias with PBLUP did not differ from its corresponding value with ssGBLUP, and in many cases it was not different from zero. The largest level bias when the subsequent evaluations were done with PBLUP was -0.18 additive genetic SD units (i.e. in the VGP scenario for ADGL in the dam line when records on animals in the validation generation were included in the subsequent evaluation; Table 6). Regression coefficients of PYD on (G)EBV in most instances did not differ between PBLUP and ssGBLUP, or from their expected value of 0.5. However, with PBLUP, the regression coefficients were sometimes larger than 0.5 (e.g. in the reference and VGP scenarios for ADGL in the sire line, and in the reference and VGP scenarios for loin depth in the dam line). In some cases, the regression coefficients were also higher with PBLUP than with ssGBLUP (e.g. in the reference and VGP scenarios for ADGT in the sire line, and in the reference and VGP scenarios for ADGL in both lines). The regression coefficients with PBLUP were lower in the MGP scenarios than in the reference scenarios for ADGT and ADGL in the dam line when records on animals in the validation generation were included in the subsequent evaluations (Table 6), and this contrasts with the regression coefficients with ssGBLUP, which did not differ between the corresponding GPS scenarios (Tables 3, 4).

## Discussion

In this study, we investigated the impact of genomic preselection (GPS) on accuracy and bias in subsequent ssGBLUP evaluations of preselected animals. We used data on production traits from one sire line and one dam line from a commercial pig breeding program in which preselection had taken place, and retrospectively implemented additional layers of GPS. For each line, we implemented three GPS scenarios and used precorrected phenotypes as records in the subsequent genetic evaluations, and progeny yield deviation (PYD) as the proxy for TBV. We conducted the subsequent genetic evaluations either with or without records on animals in the validation generation, and in all cases without progeny of validation animals. Validation accuracy decreased, or at least tended to decrease, with additional layers of GPS. Dispersion bias was largely absent, and the regression coefficient of PYD on GEBV—the indicator of dispersion bias—in all instances did not differ between corresponding GPS scenarios. Level bias was also largely absent, and mean PYD minus mean GEBV—the indicator of level bias—in most instances did not differ between GPS scenarios. The above results were observed in both lines, for all traits, and regardless of whether records on animals in the validation generation were included or not in the subsequent ssGBLUP evaluations.

Empirically assessing the impact of preselection on subsequent genetic evaluations of preselected animals requires comparison of scenarios that take different approaches into account, including scenarios without preselection. Since some GPS had already taken place in the dataset that we used for this study, it was not possible to have a scenario without preselection. Thus, we had to find an alternate way to investigate whether ssGBLUP is able to estimate GEBV in the subsequent evaluation of preselected animals without preselection bias in our current dataset and by extension in real breeding programs. We hypothesized that if ssGBLUP in subsequent evaluations yields unbiased GEBV for preselected animals in spite of additional GPS in the current dataset (i.e. in our VGP and MGP scenarios), then it also yields unbiased GEBV for preselected animals in the subsequent evaluations with the current dataset (i.e. in our reference scenarios). This is why we developed the VGP and MGP scenarios that implemented additional layers of GPS over the regular GPS that had already been implemented in the commercial pig breeding program. While scenarios such as VGP and MGP do not occur in real breeding programs, implementing these GPS scenarios in our study enabled us to investigate the impact of GPS on subsequent genetic evaluations of preselected animals using real data, by including different amounts of pedigree, genomic and phenotypic information in the subsequent genetic evaluations. Our results show that ssGBLUP in subsequent evaluations of pigs can estimate GEBV of preselected animals without preselection bias. We believe that the findings of the current study can be extended to other animal breeding programs because preselection has similar effects, regardless of its type and intensity and in how many stages it is implemented, i.e. preselection ensures that only better-than-average animals are phenotyped for the traits measured at advanced stages of the life of animals. Our findings are in line with those from our previous studies that used simulated datasets, i.e. that ssGBLUP in subsequent evaluations estimates unbiased GEBV for preselected animals, regardless of the preselection type and intensity [3, 4]. In [3], we showed that, compared to scenarios without preselection, ssGBLUP in subsequent evaluations estimated GEBV of preselected animals with a loss in accuracy, which we attributed to the smaller number of sibs with records compared to the scenarios without preselection. In the current study, accuracy also decreased, or at least tended to decrease, with additional layers of preselection, which can also be attributed to the smaller number of relatives with records compared to the scenarios with less preselection, as shown in Table 1.

The preselection that we implemented in this study and in [3] and [4] are forms of non-ignorable selection (e.g. [10, 32–34]). Without genomic information, all information used at these preselection stages must be included in subsequent evaluations to avoid preselection bias (e.g. [10, 13, 35, 36]). However, in our previous studies [3, 4], we showed that ssGBLUP in subsequent evaluations results in GEBV of preselected animals without preselection bias even if the genotypes of preculled animals are not included. In [4], we also showed that genotypes of preculled animals are only needed in the subsequent ssGBLUP evaluations if their parents are not genotyped. Again in [4], we suggested that ssGBLUP uses the genotypes of preselected animals and their parents to estimate the on average positive Mendelian sampling (MS) terms of the preselected animals, and this enables ssGBLUP in subsequent evaluations to estimate GEBV of preselected animals without preselection bias. In the current study, preselected animals and their parents were genotyped, and we indeed did not observe preselection bias in our subsequent ssGBLUP evaluations although the genotypes of preculled animals were not included in the subsequent evaluations.

### Comparison of results between preselection scenarios and between ssGBLUP and PBLUP

Our results show that, for all traits and especially for ADGT and ADGL, which were highly weighted in the breeding goal and for which GPS was highly effective, the heritability estimates increased or at least tended to increase, with additional layers of GPS. This is because changes in residual variances were larger than the corresponding changes in additive genetic variances from the reference to the VGP and to the MGP scenarios (see Additional file 2: Table S2 and Additional file 3: Table S3). The likely explanation for this observation is that some animals may have low phenotypic records for non-genetic reasons such as injury, social stress or illness, and this ‘dilutes’ the heritability. Typically, such poor-performing animals are not selected, which results in selected groups of animals being more homogeneous in terms of expressing their genetic potentials than unselected groups. This suggests the presence of heterogeneity of residual variances across herd-year classes for all traits, with the greatest heterogeneity in the reference scenarios. For both lines, we repeated the subsequent evaluations of the reference scenarios when records on animals in the validation generation were included, with a model that corrects for heterogenous residual variances (results not shown; [21]). Since we found that estimates of accuracy and bias in these scenarios did not differ between our two models, we decided to continue with the simpler model (i.e. the model in Eq. 3).

Our results also show that with both ssGBLUP and PBLUP, validation accuracy decreased or at least tended to decrease, with additional layers of GPS, which can be explained by the fact that the amount of phenotypic information also decreased in the same way (Table 1). The observation that heritabilities tended to increase with more preselection could have influenced, at least partly, the magnitudes of decreases in accuracies with decreases in amounts of phenotypic information due to preselection. This can contribute to explaining why decreases in validation accuracies with additional layers of GPS were in most cases not statistically significant. Although we always observed that the corresponding validation accuracies tended to be higher with ssGBLUP than with PBLUP, in most cases, these differences were not statistically significant. The fact that heritabilities were all relatively high (ranging from 0.24 to 0.58, Tables 3, 4, 5, 6) explains, at least partly, the absence of significant differences between ssGBLUP and PBLUP evaluations when records on animals in the validation generation were included in the subsequent genetic evaluations. It is common knowledge that the higher the heritability, the greater the importance of own performance information and the lesser the importance of genomic information in genetic evaluations (e.g. [18]).

In this study, we have shown that level bias was absent in most instances, and even when it was present it remained marginal. We have also shown that, in most cases, the measure of level bias (i.e. the difference between mean PYD and mean (G)EBV among validation animals) did not differ between corresponding GPS scenarios, regardless of whether ssGBLUP or PBLUP were used for the subsequent evaluations. Although we have not been able to find a definite explanation for the relatively small level bias observed in some scenarios in this study, we have reasons to believe that it was not caused by preselection. First, based on how we calculated level bias (i.e. as the mean progeny yield deviation minus the mean (G)EBV), level bias caused by preselection is expected to be positive, since unaccounted preselection underestimates MS terms and by extension breeding values of preselected animals, as shown by [1, 3, 4, 11, 12]. Second, level bias caused by preselection is expected to increase from the reference to the VGP and to the MGP scenarios. However, Tables 3, 4, 5, 6 show that the direction of change in level bias was not constant. In many cases, level bias was lower in the MGP scenario than in the reference and/or VGP scenarios. In our previous study [3], we observed no level bias when ssGBLUP was used in subsequent genetic evaluations, regardless of the type or intensity of preselection. However, in [3], we found that level bias increased with increasing intensity of preselection when we used PBLUP in subsequent genetic evaluations. Patry et al. [1, 11, 12] also reported significant level bias when subsequent genetic evaluations of genomically preselected animals were done with PBLUP, except when some pseudo-phenotypic information on preculled animals was included in the subsequent PBLUP evaluations. As for level bias, we found that dispersion bias was absent in most cases, regardless of whether ssGBLUP or PBLUP was used for subsequent evaluations. In our previous study with a simulated dataset [3], we found that regression coefficients of TBV on (G)EBV—the indicator of dispersion bias—were higher and closer to the expected value of 1 when ssGBLUP was used for subsequent genetic evaluations than when PBLUP was used. In [3], we also showed that the regression coefficient decreased as preselection intensity increased when PBLUP was used, but did not change when ssGBLUP was used. Preselection and subsequent selection were multi-trait in the current study, and single trait in the previous studies [1, 3, 11, 12], which means that there was more chance of having multiple litter mates remaining in the dataset after preselection and subsequent selection in the current study than in the previous studies. With multiple litter mates with records in the dataset, MS terms can be estimated reasonably well even without genomic information. This is likely the reason we did not observe level or dispersion bias in the subsequent PBLUP evaluations of preselected animals in the current study.

Without selection, the expectation of the regression coefficient of PYD on (G)EBV is 0.5, because PYD only represents half of the breeding value of the parent. However, when validation animals are on average genetically better than a random sample of their age group, the expectation of the regression coefficient decreases in single-trait subsequent evaluations, depending on how much the validation animals deviate from the average of their age group (e.g. [37, 38]). In the data used here, ADGT and ADGL had greater weights in the breeding goals of the two lines than backfat thickness and loin depth, so we expected that our GPS would have a smaller impact on the regression coefficients for backfat thickness and loin depth than for ADGT and ADGL. However, we did not observe smaller regression coefficients or regression coefficients that were further away from 0.5 for ADGT and ADGL than for backfat thickness and loin depth, either with ssGBLUP or with PBLUP. As explained in the previous paragraph, the fact that we implemented multi-trait subsequent evaluations in the current study, as opposed to the single-trait subsequent evaluations in [37] and [38], could explain the differences between these two groups of studies.

### Comparison of results between the two lines

Even in the dam line where the original GPS was at least numerically more intense and the ratio of males with records to females with records in any generation was about 20:80, in general, we observed no significant decrease in validation accuracy, or significant increase in level or dispersion bias across our GPS scenarios. However, we found that the corresponding validation accuracies for ADGT, ADGL and backfat thickness were always higher in the sire line than in the dam line, although the corresponding heritability estimates for ADGT and ADGL were in many cases higher in the dam line than in the sire line. These higher accuracies in the sire line than in the dam line may be explained by the relatively higher phenotyping and genotyping rates in the sire line than in the dam line (Table 1), meaning that validation animals in the sire line had more relatives with phenotypes and/or genotypes than validation animals in the dam lines.

### Genotypes of preculled animals did not affect the subsequent ssGBLUP evaluations

For the subsequent ssGBLUP evaluations without records on animals in the validation generation, results from the corresponding reference and VGP scenarios are exactly the same, at least up to two decimals (Tables 3, 4). However, in terms of data content, the reference scenarios contained genotypes of the animals that were preculled in the corresponding VGP scenarios, in addition to all data used in the corresponding VGP scenarios (Table 1). The fact that the results from these two scenarios are the same, means that the genotypes of the preculled animals did not affect the reference scenarios. In this study, most (about 95%) of the validation animals and their parents had genotypes. This is in line with the conclusion from our previous study [4], that genotypes of preculled animals are only useful in subsequent ssGBLUP evaluations of their preselected sibs when their parents are not genotyped.

### Potential additional sources of bias in ssGBLUP from our data

In real datasets as used here, it is difficult to completely rule out mistakes in pedigree recording and in genotyping. In the genomic data quality control stage in the current study, genotypes of a few thousand animals were discarded because the animals did not meet the genomic data quality standard (i.e. genotyped for at least 90% of the SNPs). This can, however, not completely rule out genotyping mistakes in the genomic data that passed quality control. Tables 3, 4, 5 and 6 show that, for some traits, heritabilities were different between the implemented GPS scenarios, although the animals in the base generation were the same. This implies that different subsets of the same data gave rise to different estimated (co)variance components in the base generation, and it is likely that, after implementation of some of the GPS scenarios, the estimated (co)variance components were different from their true values, at least for some of the traits. While these are all potential additional sources of bias in the ssGBLUP evaluations, they are difficult to avoid in practice [14]. However, in general, we have shown that these potential additional sources of bias do not cause significant bias in our ssGBLUP evaluations, since both level and dispersion biases were in most cases absent, and even when they were present they remained marginal and in most cases, did not differ across corresponding GPS scenarios.

## Conclusions

When subsequent genetic evaluations of preselected animals are based on ssGBLUP, genomic preselection in single or multiple generations decreases realized accuracy only slightly, and hardly causes level or dispersion bias. This conclusion is expected to hold regardless of whether records on validation animals are included or not in the subsequent evaluations, and regardless of the weight of the trait in the breeding goal. Although these conclusions were derived using data from a pig breeding program, we believe that they can be generalized to other animal breeding programs, because preselection is expected to have the same effect in any animal breeding program.

## Supplementary Information

**Additional file 1: Table S1**. Estimated heritabilities (diagonal), genetic correlations (below the diagonal) and phenotypic correlations (above the diagonal) for the traits analysed in this study, using the full data from the sire line. Standard errors are in brackets.

**Additional file 2: Table S2**. Estimated additive genetic and residual variances (standard errors in bracket) in the sire line.

**Additional file 3: Table S3**. Estimated additive genetic and residual variances (standard errors in bracket) in the dam line.

## Declarations

### Availability of data and materials

The data used in the present study were provided by Topigs Norsvin, and are not publicly accessible.
